# High resolution continuous arterial spin labeling of human cerebral perfusion using a separate neck tagging RF coil

**DOI:** 10.1371/journal.pone.0215998

**Published:** 2019-04-25

**Authors:** María Guadalupe Mora Álvarez, Robert Wayne Stobbe, Christian Beaulieu

**Affiliations:** Department of Biomedical Engineering, Faculty of Medicine and Dentistry, University of Alberta, Edmonton, Alberta, Canada; Henry Ford Health System, UNITED STATES

## Abstract

For standard clinical applications, ASL images are typically acquired with 4–8 mm thick slices and 3–4 mm in-plane resolution. However, in this paper we demonstrate that high-resolution continuous arterial spin labeling (CASL) perfusion images can be acquired in a clinically relevant scan time using current MRI technology. CASL was implemented with a separate neck coil for labeling the arterial blood on a 4.7T MRI using standard axial 2D GE-EPI. Typical-resolution to high-resolution (voxels of 95, 60, 45, 27, or 7 mm^3^) images were compared for qualitative and quantitative cerebral blood flow analysis (CBF) in nine healthy volunteers (ages: 24–32 years). The highest resolution (1.5x1.5x3 = 7 mm^3^) CASL implementation yielded perfusion images with improved cortex depiction and increased cortical CBF measurements (53 ± 8 ml/100g/min), consistent with reduced partial volume averaging. The 7 mm^3^ voxel images were acquired with 6 cm brain coverage in a clinically relevant scan of 6 minutes. Improved spatial resolution facilitates CBF measurement with reduced partial volume averaging and may be valuable for the detection of perfusion deficits in small lesions and perfusion measurement in small brain regions.

## Introduction

Arterial spin labeling (ASL) measures cerebral perfusion using radiofrequency tagged blood as an endogenous contrast agent, and offers comparable perfusion deficit detection to intra-venous Gd bolus tracking in acute stroke [1–3]. In an effort to standardize ASL measurements for clinical applications, an ASL “white paper” [4] recommends 4–8 mm thick slices and 3–4 mm in-plane resolution yielding voxel volumes from 36 mm^3^ to 128 mm^3^ in 4 minutes maximum scan time (see Table 2 in Reference [4]). While ASL protocols of this sort may be effective for large lesions, they are likely inadequate for the detection of smaller regions of perfusion deficit as may be expected in transient ischemic attack or minor stroke [5]. They are also likely inadequate for the assessment of perfusion in small brain structures [6]. The need for higher spatial resolution has been recognized in two recent ASL stroke studies at 3T that used smaller voxels of 27 mm^3^ (i.e. 3 mm isotropic) and 23 mm^3^ [7,8].

Stronger magnetic fields yield greater SNR and longer lasting arterial tags which facilitate higher spatial resolution imaging. A pseudo-continuous ASL study at 7T acquired 1.5x1.5x3 ≈ 7 mm^3^ voxels in 7 min [9], and a pulsed ASL study at 9.4T acquired 1x1x2 = 2 mm^3^ voxels in 12 min [10]. Both showed more accurate depiction of the cortex over multiple axial slices in healthy subjects. Feasibility of ~7 mm^3^ Turbo-FLASH pCASL was also demonstrated in a study at 7T [11]. However, Implementation of pulsed or pseudo-continuous ASL (i.e. PASL, pCASL) at high field can be challenging due to B_1_ inhomogeneity, off-resonance effects, and specific absorption rate (SAR) constraints [10,12]. This is particularly the case when head RF coils without neck irradiating elements are used for B_1_ transmit. In this case the creation of efficient arterial spin labeling can lead to excessive power deposition in the head. A ‘SAR-friendly’ alternative, which is also potentially insensitive to off-resonance effects, is to use a separate neck coil for labeling in combination with continuous low-power RF irradiation (CASL) [13–17]. The purpose of this work was to show that good quality high spatial resolution perfusion images are feasible in clinically relevant scan times with the use of CASL and a neck labeling coil at 4.7T.

## Materials and methods

**Fig 1:**
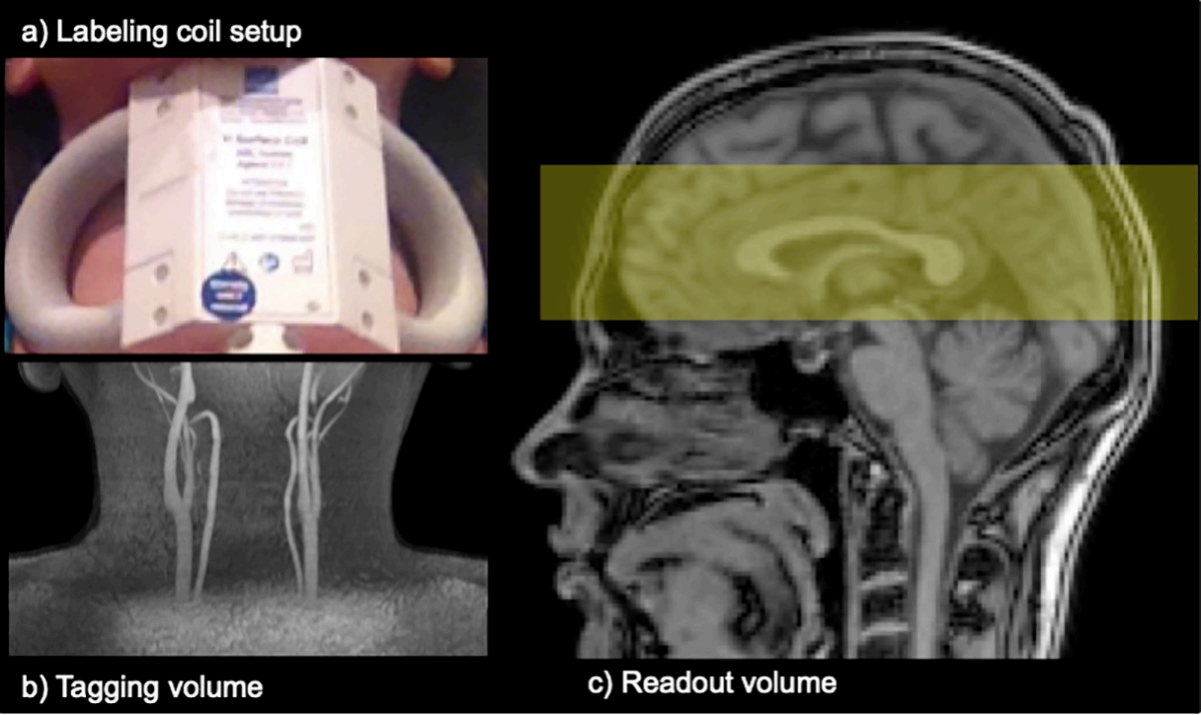
CASL neck labeling coil setup at 4.7T. a) The CASL labeling coil is placed on the neck. b) This figure shows the carotid arteries where the inflowing blood is tagged. c) The yellow area in the sagittal image shows the readout volume.

The review board of our institution approved this study, and written informed consent was obtained from all volunteers who participated. Perfusion imaging was performed on a 4.7T scanner (Varian, Walnut Creek, CA) using a butterfly neck coil for continuous arterial spin labeling with housing dimensions: length 110 mm, width 150 mm, and height 75 mm (**Fig 1)** (Rapid Biomedical, Rimpar, Germany). This coil weighed 350 g and was designed to handle a maximum mean power of 5W. Active decoupling enabled isolation from the transmit coil and receive-array during imaging. A dedicated amplifier (Communication Power Corporation, Hauppauge, New York, USA) capable of operation in continuous-wave mode provided RF power. The RF coil used for head excitation was a 16-leg detunable quadrature birdcage design with a 27 cm diameter and 25 cm leg lengths (XL-Resonance, London, Ontario, Canada) [18]. It also possessed an integral RF shield with 35.5 cm diameter. The RF coil used for reception had 4 detunable elements closely fitting the average adult head, and each element was connected to a low impedance, low noise-figure preamplifier (Pulsteq Ltd, Guildford, Surrey, UK). The irradiation range of the labeling coil was tested to be outside the brain and the transmit and receive coils were detuned during the labeling period. Thus, magnetization transfer effects in brain were avoided, a known advantage of separate labeling coils [16,17,19].

For arterial spin labeling, RF irradiation (1.4 W) was applied to the neck coil in the presence of a G_Z_ = 2.5 mT/m gradient. A frequency offset of ~18 kHz (depending on the volunteer) localized the flow driven adiabatic blood excitation around the centre of the neck coil. The labeling duration was τ = 3 seconds, and the post labeling delay was PLD = 1500 ms [20]. Note that these parameters were selected according to previous CASL studies [13–16,21] and internal optimization. The effective volume irradiated by the neck coil was measured to be ~200 mL (from B_1_ mapping experiment on three volunteers) and thus the 1.4W tagging power resulted in a local time-averaged SAR in the neck of ~5 W/kg, well below the 10W/kg local SAR regulations.

For ‘control’ images no RF power was applied to the neck coil, but the gradient was left on during the tagging period to minimize changes between the tag and control sequences. Images were acquired sequentially from superior to inferior as arterial transit times are longer in the occipital lobe [22]. To measure the labeling efficiency (α), 1.5x1.5x5 mm^3^ images were acquired 5 cm above the neck coil centre from 3 healthy young volunteers. The change in signal intensity (ΔSI) between control (SI_control_) and tag (SI_tag_) images was then used to calculate α = ΔSI/(2SI_control_) in the carotid arteries.

For this study, 5 different CASL images with different resolutions were acquired in the same scan session from five healthy female and four healthy male volunteers (ages 24–32 years) with 2D single-shot GE-EPI, GRAPPA R = 2, partial-Fourier 0.7, and FOV of 204x216 mm^2^. The perfusion sequences tested were: (1) 3.5x3.4x8 = 95 mm^3^, TR 4.6 seconds, TE 6.2 ms; (2) 3.5x3.4x5 = 60 mm^3^, TR 4.6 seconds, TE 6.2 ms; (3) 3x3x5 = 45 mm^3^, TR 4.6 seconds, TE 8 ms; (4) 3x3x3 = 27 mm^3^, TR 4.6 seconds, TE 8 ms; and (5) 1.5x1.5x3 = 7 mm^3^, TR 4.7 seconds, TE 13.2 ms. Each scan was 5 minutes for 30 tag and 30 control interleaves, and five axial slices were acquired in each case. A 4 mm inter-slice gap was chosen to minimize the cross-talk effect for the thickest 8 mm slices, and this (more than necessary with respect to cross-talk) gap retained for the scans with thinner slices to approximately match regions of brain imaged between scans. Note that due to time limitations low-resolution images (95 mm^3^ and 60 mm^3^) were not acquired from two male volunteers.

Image processing and analysis first involved motion correction across all averages using rigid body transformation (in-house software in Matlab). For each scan averaged tag images were then subtracted from the averaged control images to obtain the difference images reflecting perfusion. The change in signal intensity (ΔSI) was measured in the cortical gray matter and white matter over all five slices using masks that were manually drawn on the control acquisitions (note that separate anatomical images for tissue segmentation were not acquired due to scan-time constraints). Finally, high-resolution (1.5x1.5x3 = 7 mm^3^, TR 5.1 seconds, TE 13.2 ms) images were acquired from 6 healthy volunteers (ages: 24–39 years) in 6 minutes with 15 slices and 1 mm inter-slice gap to test the acquisition of a reasonable 6 cm brain coverage.

CBF was quantified using Buxton’s General Kinetic Model [23] as recommended by [4] using the following parameters: labeling duration (τ) = 3 s, labeling efficiency (α) = 0.83, blood-brain partition coefficient (*λ*) = 0.9 mL/g [24], PLD = 1.5 s (plus an additional delay for each subsequent slice), T_1blood_ at 4.7T = 2004 ms [25], and T_1tissue_ at 4.7T = 1630 ms [26] (**Eq 1**).

$$CBF=\frac{6000∙\lambda ∙({SI}_{control}-{SI}_{tag})∙e^{\frac{PLD}{T_{1blood}}}}{2∙\alpha ∙T_{1,blood}∙\frac{{SI}_{control}}{1-e^{-\frac{TR}{T_{1tissue}}}}∙(1-e^{-\frac{\tau }{T_{1blood}}})}\;\frac{\frac{ml}{100g}}{\min}$$ Eq 1

## Results

The labeling efficiency of arterial blood tagging at 4.7T with the butterfly neck coil and continuously applied RF was measured to be 0.83, 0.81, and 0.85 in the carotid arteries of 3 volunteers.

**Fig 2:**
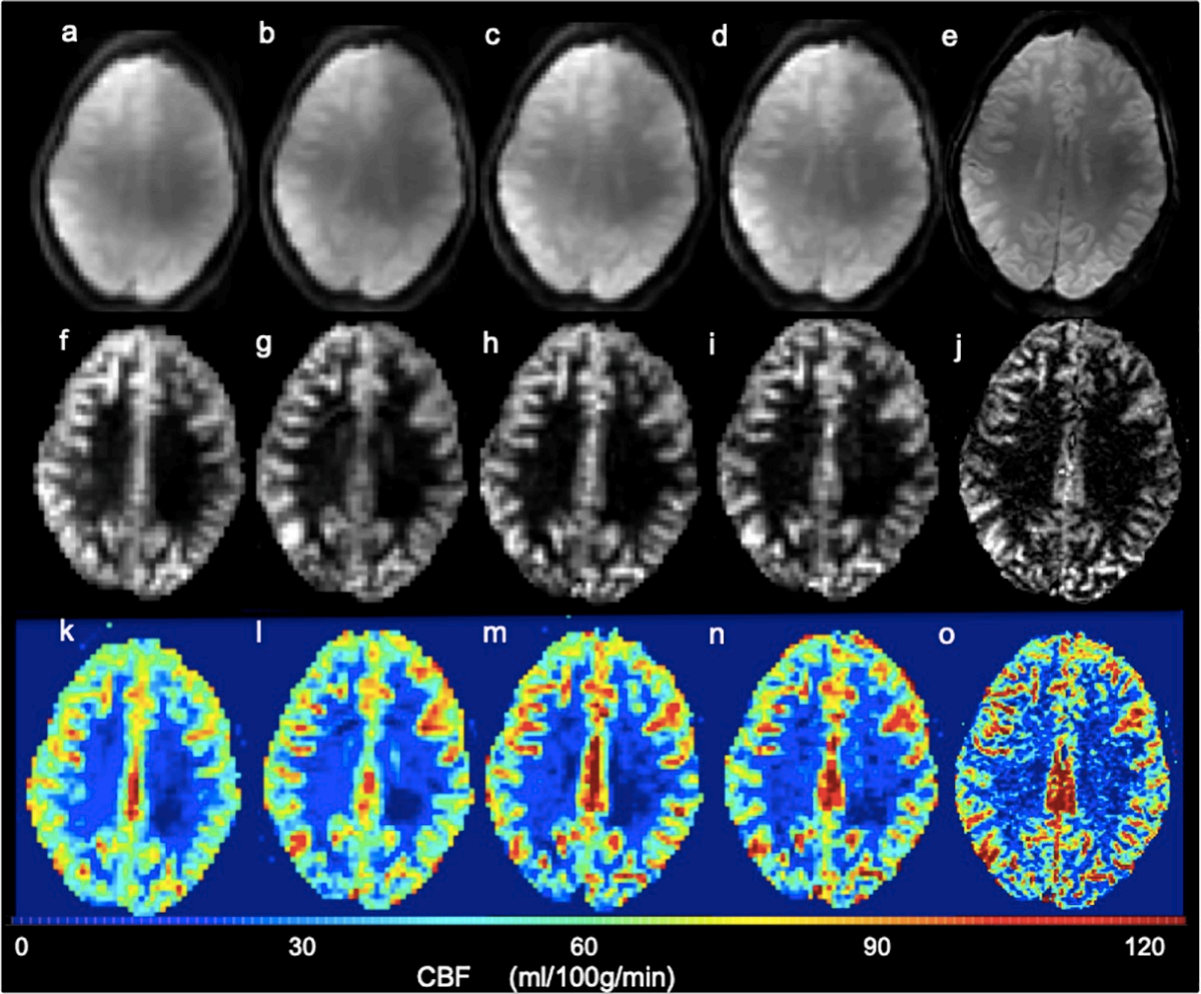
**Example raw control images (a-e), perfusion (|SI**_**control**_−**SI**_**tag**_|**) (f-j) and CBF maps (k-o) for one slice in a 25 year old healthy female at five different resolutions: (a, f, k) 3.5x3.4x8 = 95 mm**^**3**^**, (b, g, l) 3.5x3.4x5 = 60 mm**^**3**^**, (c, h, m) 3x3x5 = 45 mm**^**3**^**, (d, i, n) 3x3x3 = 27 mm**^**3**^**, and (e, j, o) 1.5x1.5x3 = 7 mm**^**3**^. Increased spatial resolution yields improved perfusion depiction as well as increased CBF values in the cortex. Note that the qualitative perfusion images (f-j) are biased by coil sensitivity, but the CBF maps (k-o) which include division by SI_control_ have this bias removed.

**Fig 3:**
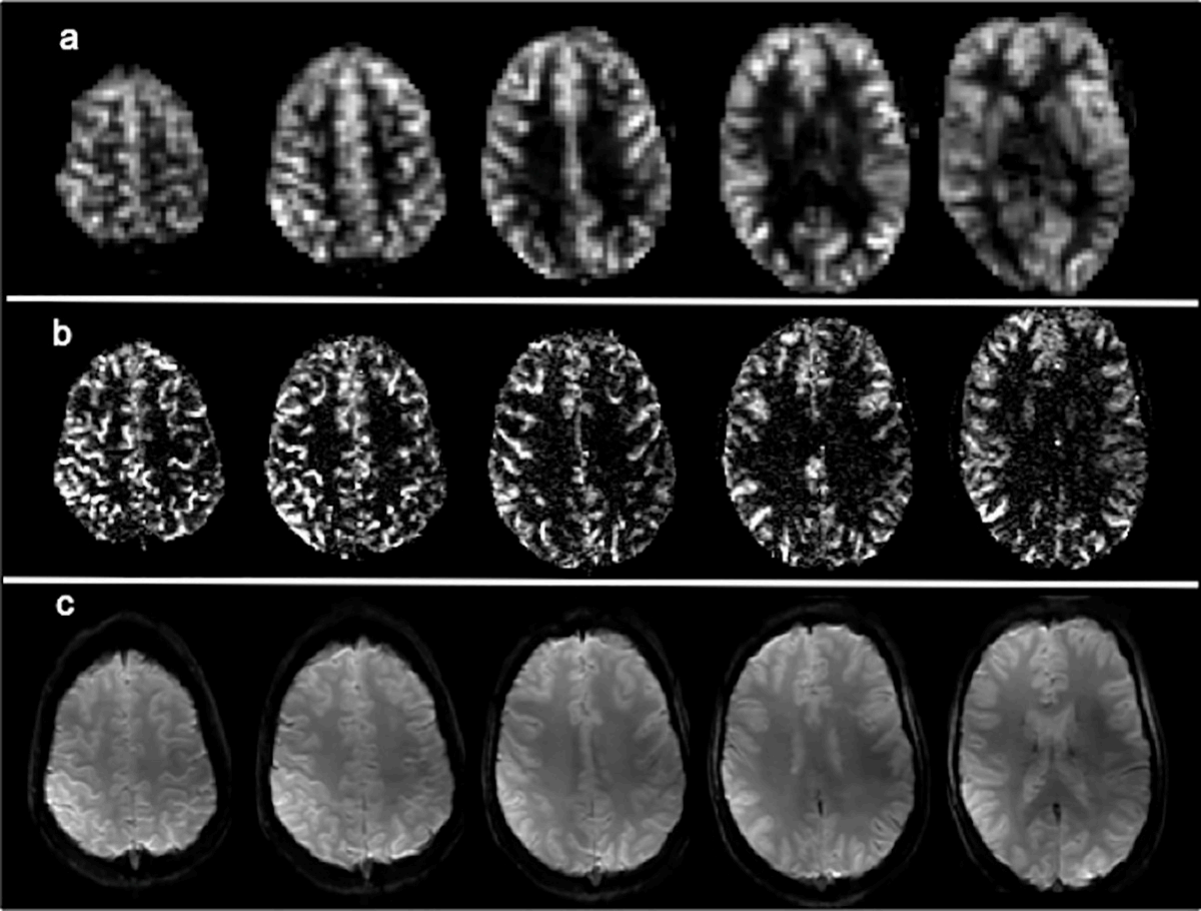
**Comparison between the low (95 mm**^**3**^**, a) and high resolution (7 mm**^**3**^**, b) perfusion images of 5 axial slices in a female volunteer (age 29).** The cortex is better depicted in the higher voxel resolution scans, which has been improved ~ 14 times relative to the conventional ASL resolution. High-resolution raw control images (c) are provided as a reference for anatomical structures.

**Fig 4:**
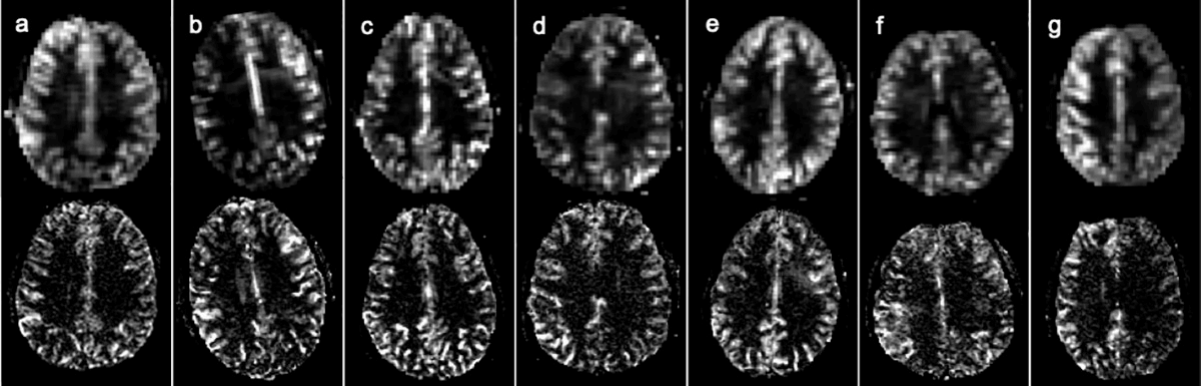
Comparison between “typical” (upper row, 60 mm^3^) and high resolution (lower row, 7 mm^3^) perfusion images of a similar slice in the other 7 healthy subjects, showing the reproducibility of CASL with a separate neck coil.

Base 2D GE-EPI images, qualitative perfusion images (|SI_control_−SI_tag_|), and CBF maps at 4.7T are shown for a slice of one healthy volunteer (age 25 years) at five different resolutions: 3.5x3.4x8 = 95 mm^3^, 3.5x3.4x5 = 60 mm^3^, 3x3x5 = 45 mm^3^, 3x3x3 = 27 mm^3^, and 1.5x1.5x3 = 7 mm^3^ (**Fig 2**). As expected, the cortex is much better depicted in the high-resolution scans. The advantage of high-resolution is also evident over multiple slices from another volunteer (**Fig 3**), and excellent perfusion depiction in the cortex was consistently observed for the other seven healthy volunteers as well (**Fig 4**).

The proportional change in signal intensity between tag and control scans (ΔSI/SI_control_) in gray matter falls in a narrow range (from 0.8% of control image intensity for low resolution to 1.2% for high resolution) and is clearly greater than in white matter (~0.3% of control image intensity across image resolutions) (**Table 1**). Calculated mean CBF in cortical gray matter (GM) was 45±7 ml/100g/min on the typical resolution scans (95 mm^3^). This CBF value increased by 20% to 53±8 ml/100g/min on the highest resolution (7 mm^3^) scans (**Table 1**). The GM/WM CBF ratios increased consistently from 2.7 to 3.5 as the resolution improved.

**Table 1:** CBF and ΔSI/SI_control_ in cortical gray matter and white matter at five different resolutions in 9 healthy volunteers (mean +/- SD).

Resolution (mm^3^)	CBF GM (ml/100g/min)	CBF WM (ml/100g/min)	CBF (GM/WM)	ΔSI/SI_control_ GM (%)	ΔSI/SI_control_ WM (%)
**3.5x3.4x8 = 95**	45 ± 7	16 ± 3	2.7	0.97 ± 0.17	0.38 ± 0.07
**3.5x3.4x5 = 60**	47 ± 6	16 ± 2	3.0	1.03 ± 0.15	0.37 ± 0.05
**3 x 3 x 5 = 45**	49 ± 7	17 ± 2	2.9	1.04 ± 0.20	0.36 ± 0.05
**3 x 3 x 3 = 27**	49 ± 6	16 ± 2	3.1	1.04 ± 0.19	0.35 ± 0.06
**1.5x1.5x3 = 7**	53 ± 8*	15 ± 4	3.5	1.11 ± 0.22	0.32 ± 0.08

CBF = cerebral blood flow; GM = Gray Matter; WM = White Matter, ΔM = proportional change of magnetization between tag/control scans; M_control_ = magnetization in control scans.

* The GM CBF at 7 mm^3^ was significantly greater than at 27 mm^3^ (p = 0.0003, one-tail paired T-tests).

**Fig 5:**
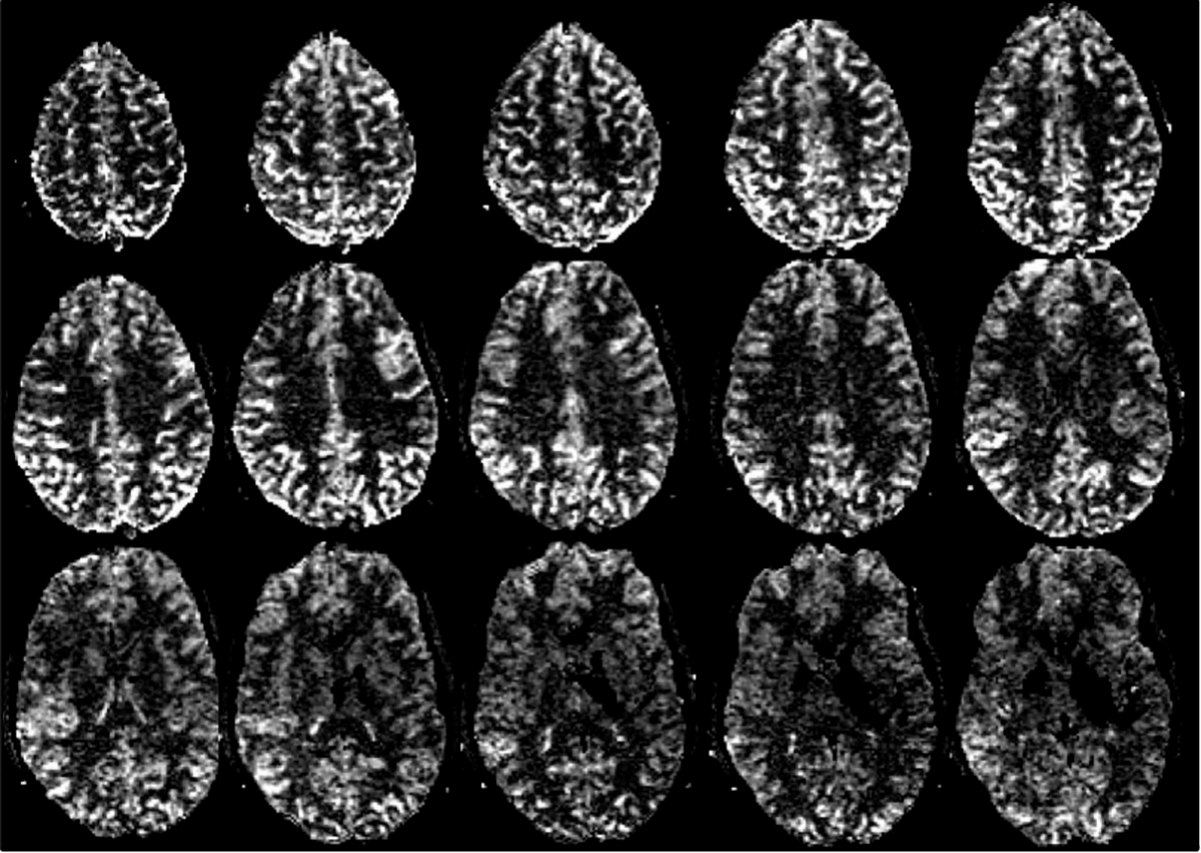
Perfusion images over 15 slices acquired with 1.5x1.5x3 mm^3^ voxels in 6 min in a healthy volunteer (age: 28 years). These high-resolution ASL images show detail in the cortical gray matter gyri. The signal becomes less in inferior slices with loss of arterial tag as the slices are acquired in order of superior (post-labelling delay, PLD 1.5 s) to inferior (PLD 2.3 s). Hyperintensities in the frontal and bilateral occipital lobes are normal findings in young and middle aged patients [27]. Hypointensity on the lower slices in the middle cerebral artery territory may be the result of tag loss, as arterial transit times are known to be shorter in this region [28] and the inferior slices are acquired last. Note that the qualitative perfusion images shown will also be biased by coil sensitivity.

Representative high-resolution (1.5x1.5x3 mm^3^) perfusion images with a clinically relevant slab coverage (6 cm with 15 3 mm slices and 1 mm gap) were acquired in 6 min. These images show particularly good quality in superior slices (**Fig 5**). Recall that the slices were acquired superior to inferior, and loss of the arterial tag with time leads to reduced image quality in the inferior slices. Note the higher perfusion visible in small deep gray matter structures such as the caudate.

## Discussion

Continuous arterial spin labeling with a separate neck coil facilitates the acquisition of high-resolution CBF images due to high perfusion contrast [29], good labeling efficiency and low SAR which is particularly important for high fields. In this study, we demonstrate that (1.5x1.5x3 mm^3^) images of cerebral blood flow are feasible in a 6 minute scan time at 4.7T. These higher-resolution images reduce partial volume effect and facilitate improved cortex depiction.

The highest resolution images in our study (7 mm^3^) yielded GM CBF values of 53±8 ml/100g/min, ~20% greater than the standard resolution images (95 mm^3^). This GM CBF increase was also shown in a previous study which compared ~19 mm^3^ with ~169 mm^3^ voxel CBF measurements [30]. This reflects lower partial volume averaging in the better-defined cortex. Note that CBF values varying from 40–100 ml/100g/min are considered normal in gray matter [4]. In healthy young adults a GM CBF of 51±7 (91 mm^3^ voxels) has been measured with CASL [31], and a GM CBF of 74 ± 11 ml/100g/min (90 mm^3^ voxels) measured with pCASL [32]. The values of GM CBF (45–53 ml/100g/min) reported in our work fit within the literature range. The ASL literature also reports GM/WM CBF ratios that vary from 1.6 [33] (31 mm^3^ voxels) to 3.2 [34] (51 mm^3^ voxels), acquired with pCASL 2D spin-echo spiral and PASL 3D-GRASE, respectively. The GM/WM CBF ratios measured in our work increase with improving resolution from 2.7 to 3.5 as a result of reduced partial volume averaging. The oxygen uptake rate is 6 ml/100g/min in the cortex and 2 ml/100g/min in white matter [35–36], and thus the GM/WM oxygen uptake ratio agrees with our cerebral blood flow ratios. Note, however, that WM CBF values are difficult to measure due to longer arterial transit times and low SNR.

CBF measurements vary across labeling techniques (e.g. different labeling efficiencies), acquisition methods (e.g. 2D or 3D), and assumptions or measurements for quantification (e.g. T1-maps). A high resolution 1.5x1.5x1.5 mm^3^ isotropic CASL study at 3T on rhesus monkeys showed variability in tagging efficiency based on differences in anatomy and tagging coil placement [37]. As our study primarily concerned investigation of relative intra-volunteer differences between low and high-resolution CASL imaging at 4.7T, labeling efficiency was not measured during the perfusion imaging scanning sessions. Rather an average value of α = 0.83 was used for CBF quantification. For each volunteer and imaging setup (e.g. neck coil placement) the labeling efficiency will vary, and thus the CBF values presented in this work will be biased by lack of inexact knowledge of α. However, for each volunteer the labeling efficiency remains constant and does not affect relative differences in measured CBF between imaging resolutions. Any variation in actual labeling efficiency will yield an inversely proportional error in CBF (**Eq 1**). T1 was assumed constant, as T1 maps were not acquired in our protocol. However, even with potential labeling efficiency and T1 variation, the CBF values over 9 healthy controls of similar age were quite similar with about a 15% standard deviation. Note that most clinical ASL studies [4,38–40] do not measure α and assume a constant T1 value as was done here. The qualitative perfusion (|SI_control_−SI_tag_|) images shown in **Figs 2–5** will be biased by B1 inhomogeneity. However, the CBF maps in **Fig 2** and the CBF values in **Table 1**have this bias removed as a result of division by SI_control_ (see **Eq 1**).

Diffusion and perfusion MRI are important diagnostic imaging methods for acute stroke, but current low-resolution protocols may be missing brain injury. Two studies in transient ischemic attack (TIA) patients with low resolution ASL (voxels of 54 and 60 mm^3^, field strength 1.5T and 1.5T/3T, respectively) have shown perfusion deficits in approximately half of the patients where diffusion-weighted imaging (DWI) is negative [41,42]. High resolution ASL may have identified perfusion anomalies and aided in confirmation of a clinical diagnosis of stroke in some of the other patients, but this remains to be studied relative to the typical low resolution ASL. Also, recent ASL studies on different neurological diseases such as epilepsy [43], glioblastoma multiforme [44], Moyamoya disease [45], and post-traumatic stress disorder [46] also used low resolution perfusion voxels (98, 45, 46, and 37 mm^3^, respectively). High-resolution ASL images may also benefit the study of small brain structures. A recent study of major depressive disorder used high-resolution ASL to study CBF in the habenula; however no differences were measured between major depressive disorder and healthy volunteers [47]. High-resolution ASL may also facilitate examination of perfusion in the hippocampus, where measurements of CBF are challenging due to its anatomy and physiological aspects [48].

A 2005 review article showed high resolution CASL (1.5x1.5x3 = 7 mm^3^, 12 slices) with a labeling RF coil at 3T using a 16-channel reception coil in 10.5 min [21], but clinical protocols have not adapted these long duration, high-resolution ASL scans. In our research, the SNR increase of higher magnetic field (4.7T) facilitated CASL with high spatial resolution (1.5x1.5x3 = 7 mm^3^) and 6 cm brain coverage (15 slices) in a shorter scan time of 6 min. Compared to previous ~5 min ASL studies at 3T [43,49], voxel resolution has been improved by 13 times with only a small scan time penalty. While 6 cm coverage does not include the full brain (~14.5 cm) this imaging window can be centered on the ischemic core to better assess local perfusion deficit in acute stroke. Improved brain coverage and consistent PLD across the brain may be facilitated by the implementation of improved k-space acquisition strategies such as 3D-GRASE [16,31]. Note that while a PLD of 1500 ms [20] was used in this study, longer values of 1800 ms– 2000 ms may be more appropriate for elderly adult and clinical populations [4,20].

A limitation of 2D EPI acquisition is that further increase in brain coverage results in loss of the arterial tag (for slices acquired last). However, positioning a set of slices around diffusion-visible lesions in the setting of acute stroke is a viable approach to assess the diffusion-perfusion mismatch. A practical limitation of CASL is reduced patient comfort given the weight and positioning of the neck labeling coil. CASL also requires a continuous-wave capable RF amplifier for the long duration tagging pulse and the capability to appropriately detune/tune the head and neck coils. Special care must be taken to keep the labeling coil fixed as close as possible to the neck, as variation in placement (e.g. tilting) could result in labeling efficiency variation between arteries. However, by taking advantage of high field and current EPI capabilities, high-resolution CASL could aid in the detection and measurement of CBF changes in smaller regions that are associated with neurological disease.

## Conclusions

High-resolution CASL images (7 mm^3^) with a separate labeling neck coil were shown to be feasible at 4.7T in 6 minutes with a 6 cm multi-slice coverage. Higher spatial resolution depicted perfusion in the cortical gray matter much better than in the typical ASL protocols and also yielded greater CBF values in line with less partial volume effects. High-resolution ASL images could facilitate improved detection and quantification of small perfusion deficits.
